# Supplementary material for: p57Kip2 is an essential regulator of vitamin D receptor-dependent mechanisms
Source: PLoS One. 2023 Feb 15;18(2):e0276838. doi: 10.1371/journal.pone.0276838 (PMC9931147; doi:10.1371/journal.pone.0276838)

Figure 1A Immunoprecipitation of mouse primary osteoblasts whole lysates
IP; anti-VDR antibody, IB; anti-p57 antibody

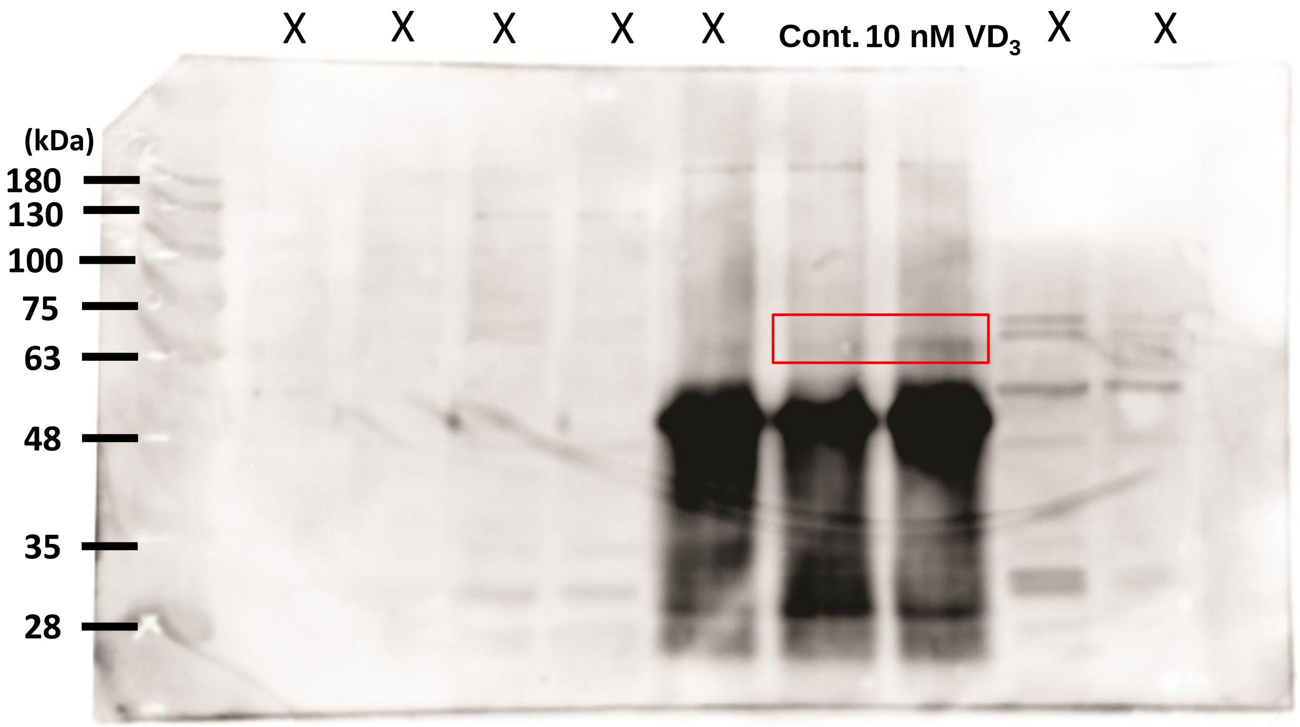

Figure 1B Immunoprecipitation of mouse primary osteoblasts whole lysates
IP; anti-VDR antibody, IB ; anti-VDR antibody

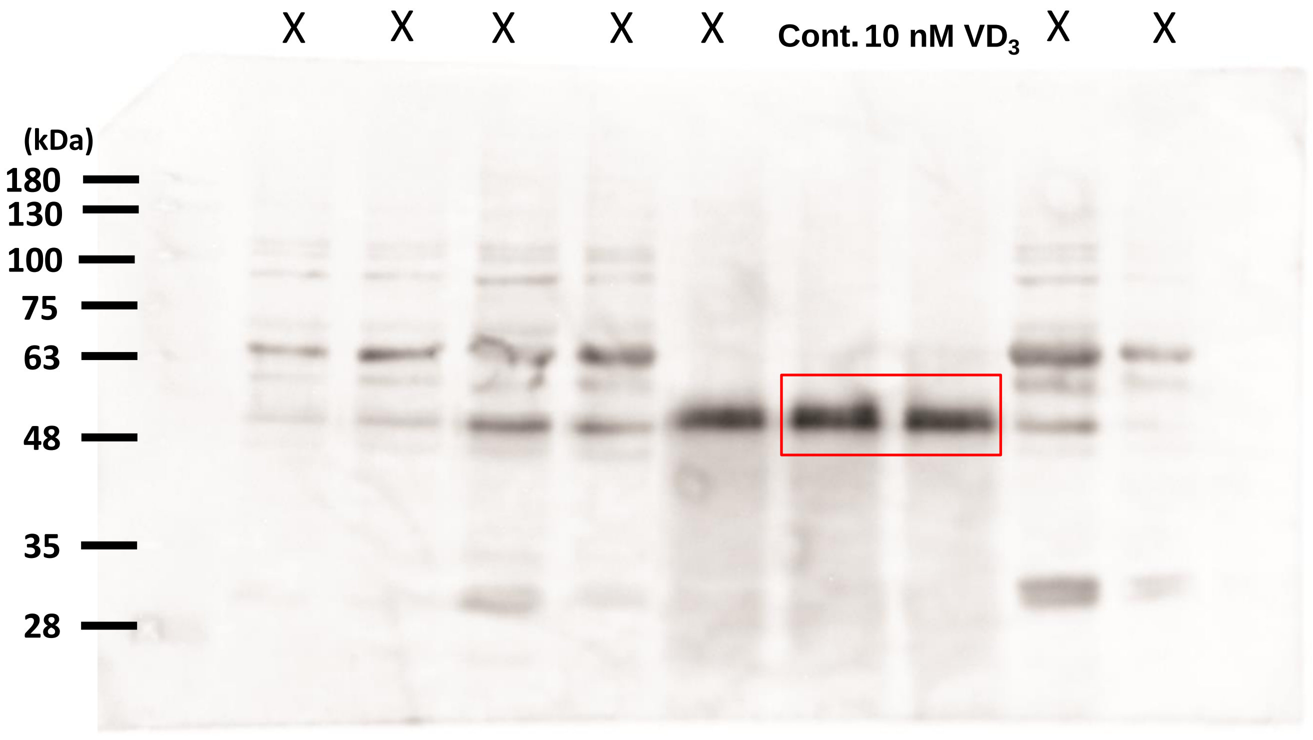

Figure 3B Detection of p57[Kip2] in mouse primary osteoblasts whole lysates

IB; anti-p57 antibody

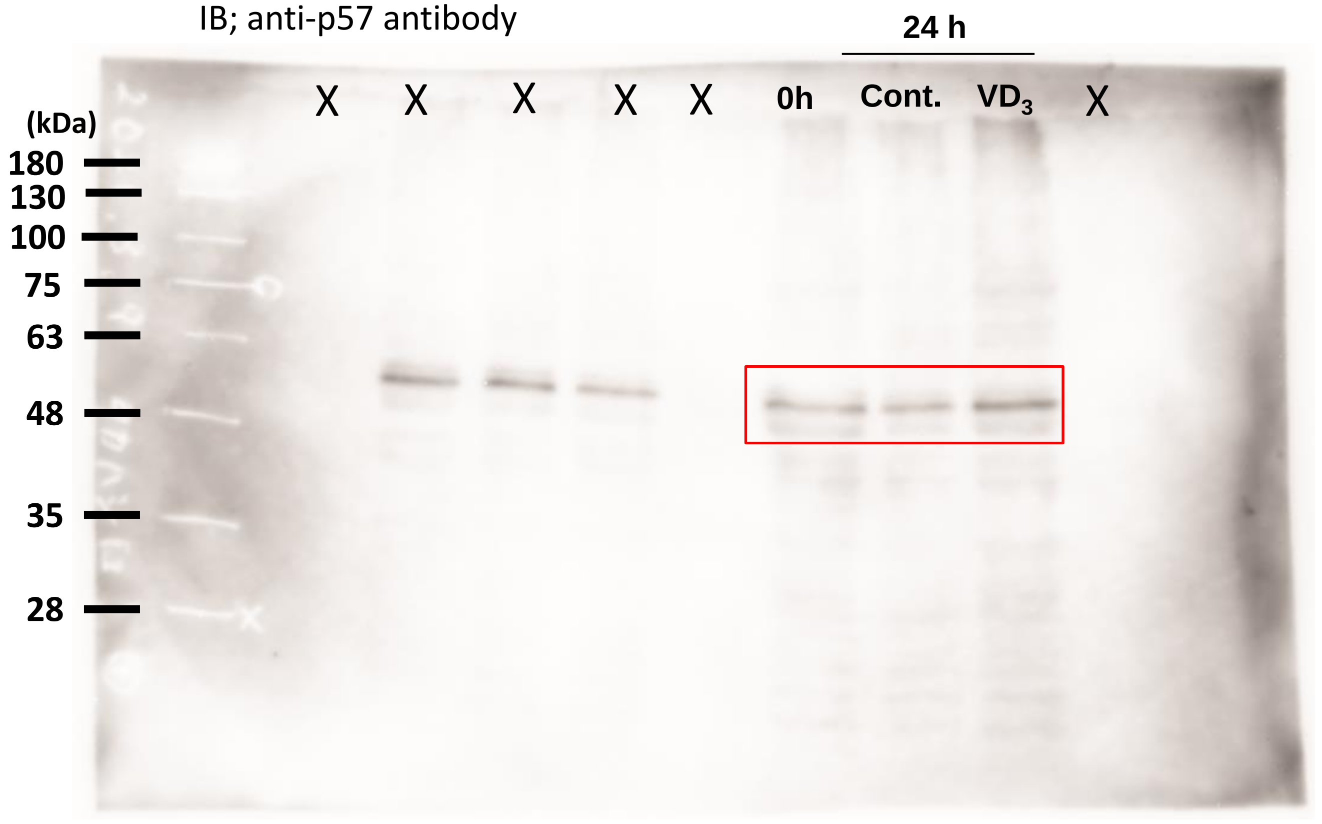

IB; anti-beta Actin antibody

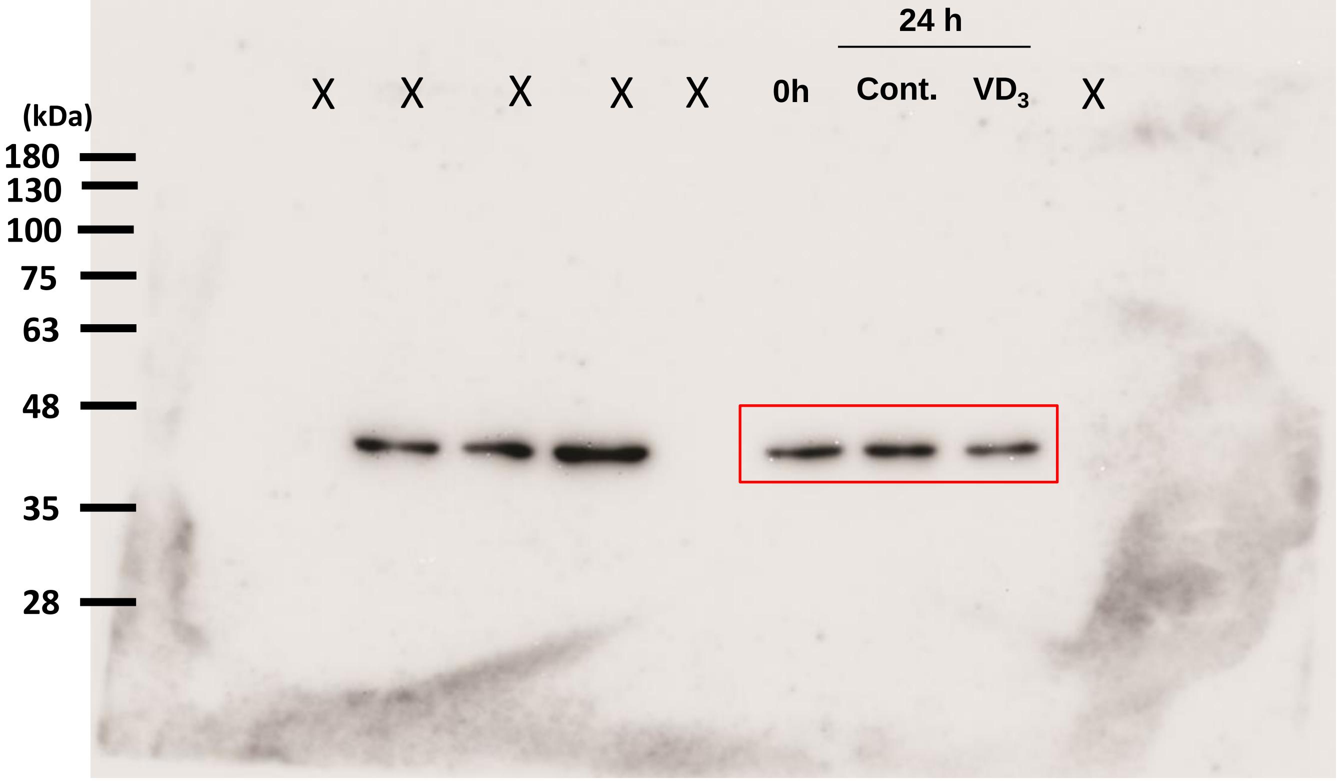

Supplemental Figure 1A

Detection of Cdks in whole lysates from *p57+/+* and *p57-/-* mouse primary osteoblasts

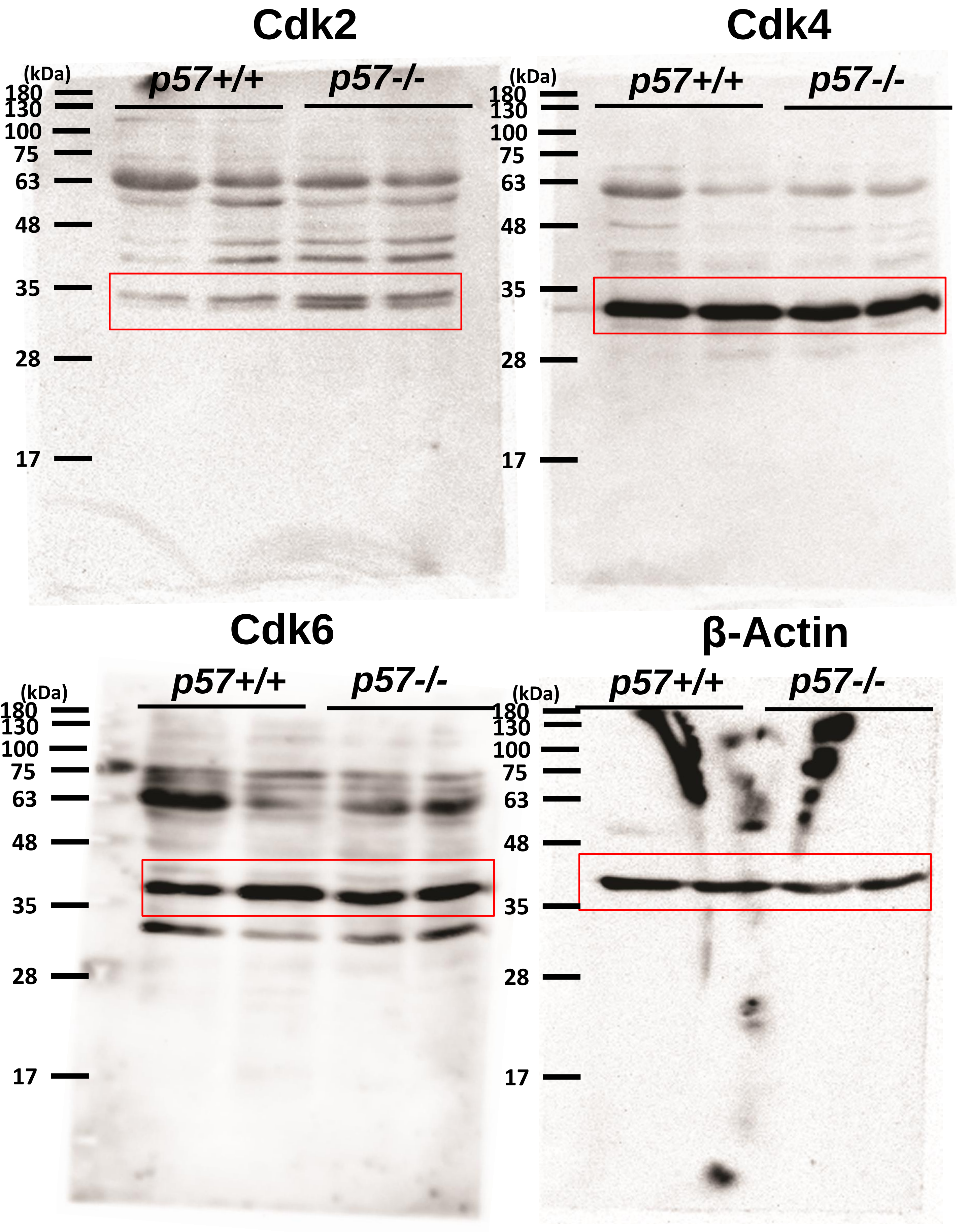

Supplement: S1 Raw images — (PDF) [file pone.0276838.s001.pdf]
