# Supplementary material for: p57Kip2 is an essential regulator of vitamin D receptor-dependent mechanisms
Source: PLoS One. 2023 Feb 15;18(2):e0276838. doi: 10.1371/journal.pone.0276838 (PMC9931147; doi:10.1371/journal.pone.0276838)
Supplement: S1 File — (PPTX) [file pone.0276838.s002.pptx]

## Slide 1
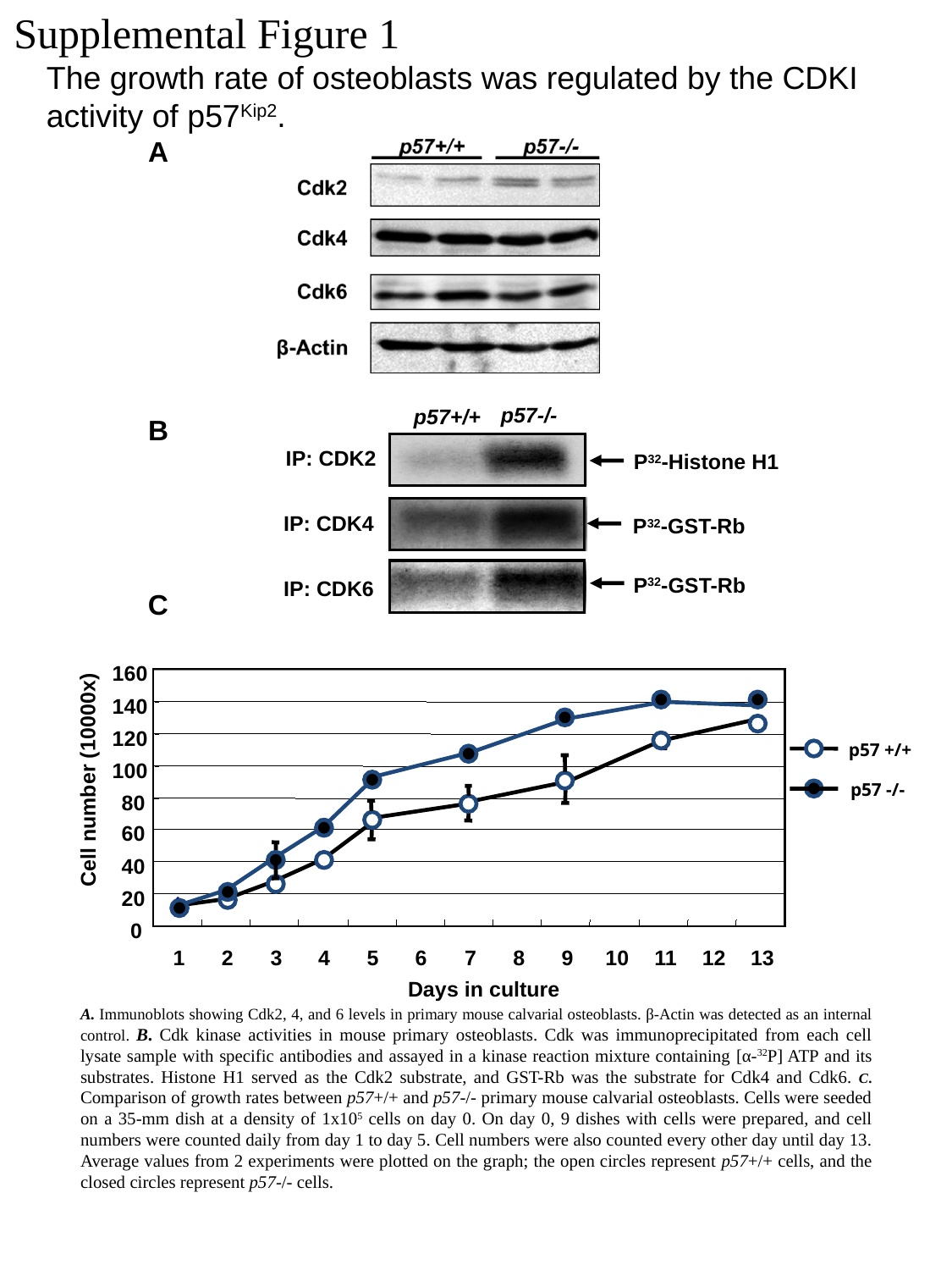

Supplemental Figure 1
The growth rate of osteoblasts was regulated by the CDKI activity of p57Kip2.
A
p57-/-
p57+/+
B
IP: CDK2
P32-Histone H1
IP: CDK4
P32-GST-Rb
P32-GST-Rb
IP: CDK6
C
160
140
120
 p57 +/+
100
Cell number (10000x)
p57 -/-
80
60
40
20
0
1
2
3
4
5
6
7
8
9
10
11
12
13
Days in culture
A. Immunoblots showing Cdk2, 4, and 6 levels in primary mouse calvarial osteoblasts. β-Actin was detected as an internal control. B. Cdk kinase activities in mouse primary osteoblasts. Cdk was immunoprecipitated from each cell lysate sample with specific antibodies and assayed in a kinase reaction mixture containing [α-32P] ATP and its substrates. Histone H1 served as the Cdk2 substrate, and GST-Rb was the substrate for Cdk4 and Cdk6. C. Comparison of growth rates between p57+/+ and p57-/- primary mouse calvarial osteoblasts. Cells were seeded on a 35-mm dish at a density of 1x10^5^ cells on day 0. On day 0, 9 dishes with cells were prepared, and cell numbers were counted daily from day 1 to day 5. Cell numbers were also counted every other day until day 13. Average values from 2 experiments were plotted on the graph; the open circles represent p57+/+ cells, and the closed circles represent p57-/- cells.

## Slide 2
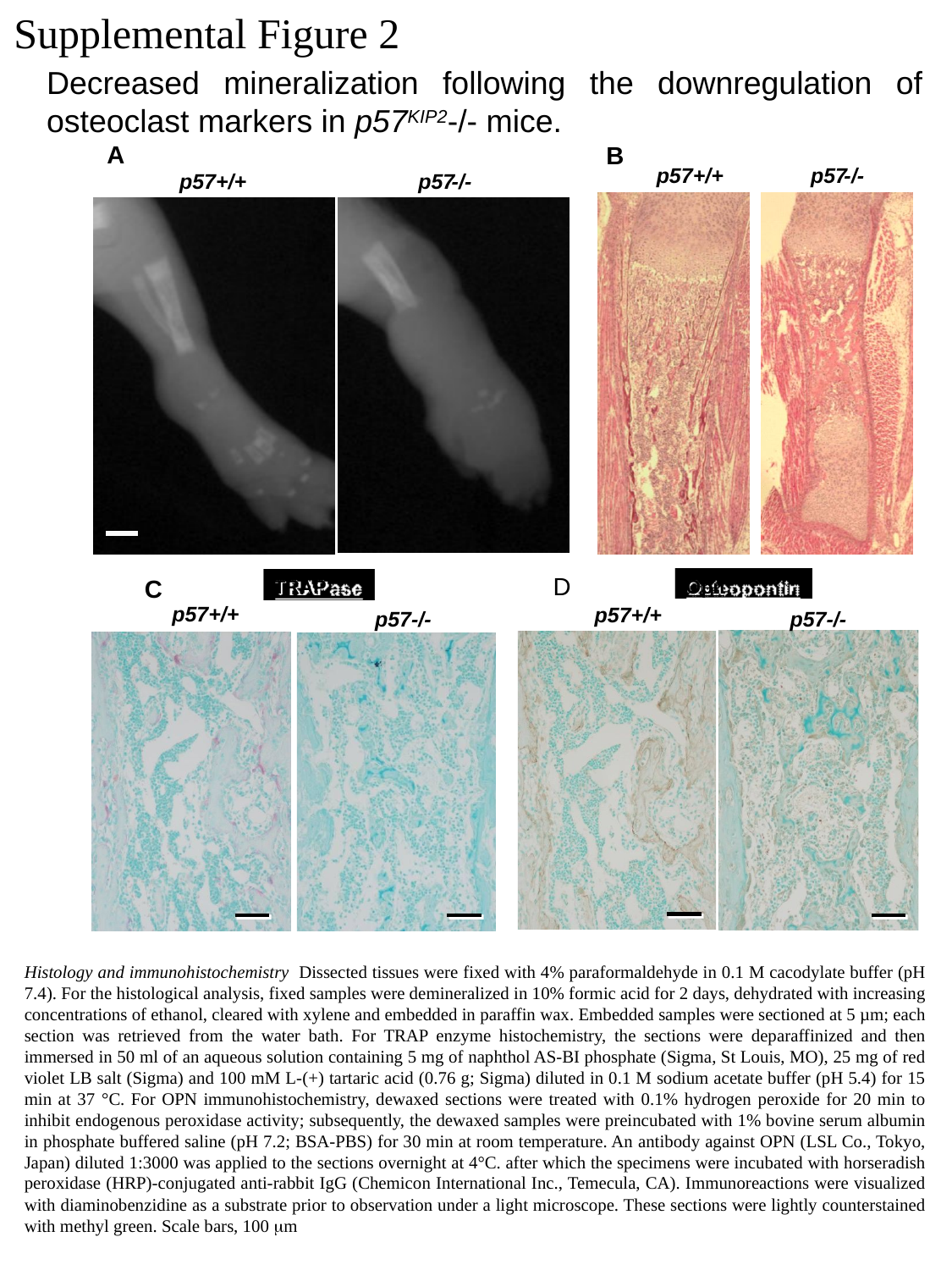

Supplemental Figure 2
Decreased mineralization following the downregulation of osteoclast markers in p57KIP2-/- mice.
A
B
p57+/+
p57
-
/
-
p57+/+
p57
-
/
-
p57+/+
D
C
TRAP
Opn
p57+/+
p57+/+
p57-/-
p57-/-
Histology and immunohistochemistry Dissected tissues were fixed with 4% paraformaldehyde in 0.1 M cacodylate buffer (pH 7.4). For the histological analysis, fixed samples were demineralized in 10% formic acid for 2 days, dehydrated with increasing concentrations of ethanol, cleared with xylene and embedded in paraffin wax. Embedded samples were sectioned at 5 µm; each section was retrieved from the water bath. For TRAP enzyme histochemistry, the sections were deparaffinized and then immersed in 50 ml of an aqueous solution containing 5 mg of naphthol AS-BI phosphate (Sigma, St Louis, MO), 25 mg of red violet LB salt (Sigma) and 100 mM L-(+) tartaric acid (0.76 g; Sigma) diluted in 0.1 M sodium acetate buffer (pH 5.4) for 15 min at 37 °C. For OPN immunohistochemistry, dewaxed sections were treated with 0.1% hydrogen peroxide for 20 min to inhibit endogenous peroxidase activity; subsequently, the dewaxed samples were preincubated with 1% bovine serum albumin in phosphate buffered saline (pH 7.2; BSA-PBS) for 30 min at room temperature. An antibody against OPN (LSL Co., Tokyo, Japan) diluted 1:3000 was applied to the sections overnight at 4°C. after which the specimens were incubated with horseradish peroxidase (HRP)-conjugated anti-rabbit IgG (Chemicon International Inc., Temecula, CA). Immunoreactions were visualized with diaminobenzidine as a substrate prior to observation under a light microscope. These sections were lightly counterstained with methyl green. Scale bars, 100 mm

## Slide 3
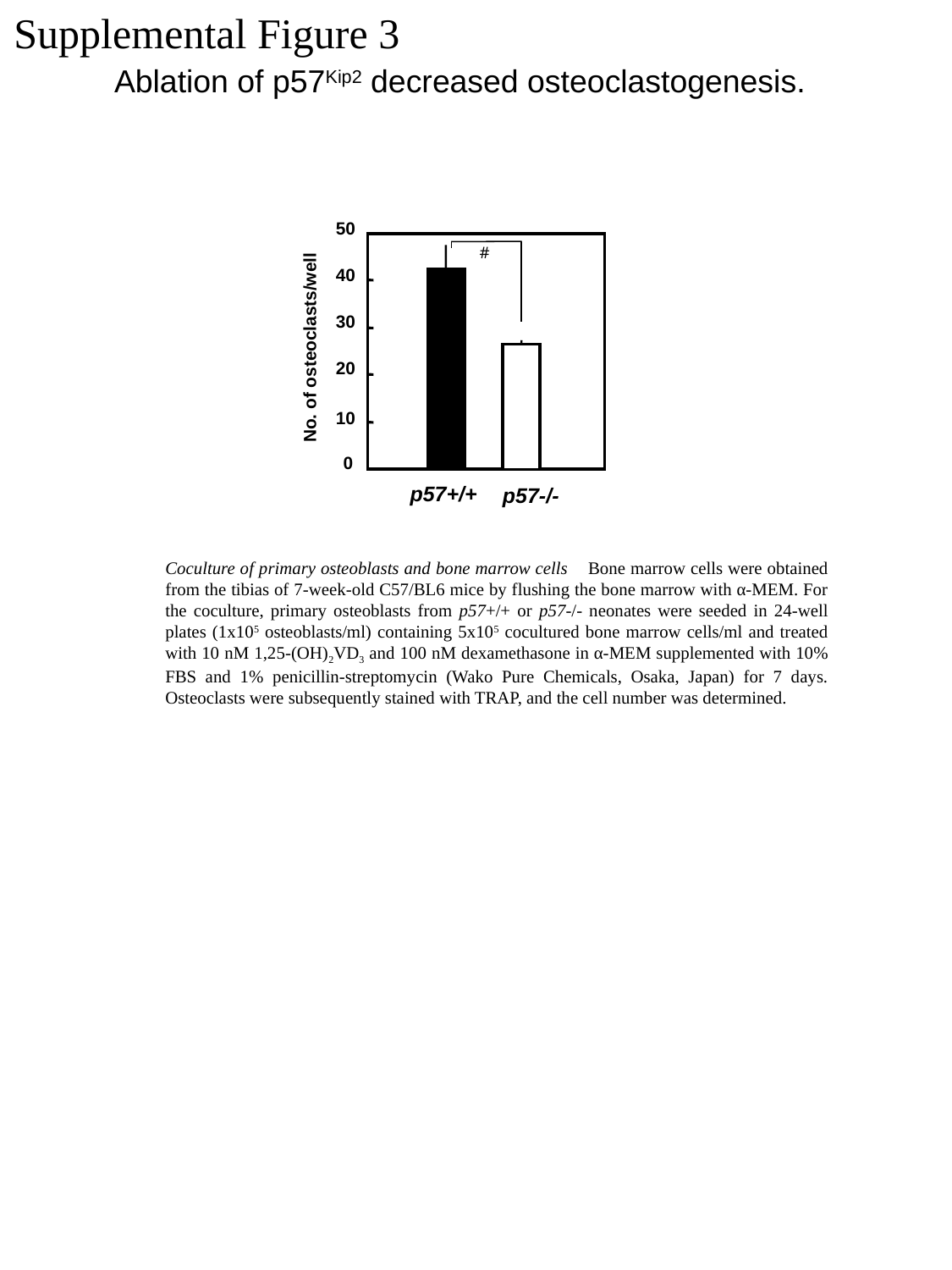

Supplemental Figure 3
Ablation of p57Kip2 decreased osteoclastogenesis.
50
40
30
No. of osteoclasts/well
20
10
0
 p57+/+
 p57-/-
#
Coculture of primary osteoblasts and bone marrow cells Bone marrow cells were obtained from the tibias of 7-week-old C57/BL6 mice by flushing the bone marrow with α-MEM. For the coculture, primary osteoblasts from p57+/+ or p57-/- neonates were seeded in 24-well plates (1x10^5^ osteoblasts/ml) containing 5x10^5^ cocultured bone marrow cells/ml and treated with 10 nM 1,25-(OH)2VD3 and 100 nM dexamethasone in α-MEM supplemented with 10% FBS and 1% penicillin-streptomycin (Wako Pure Chemicals, Osaka, Japan) for 7 days. Osteoclasts were subsequently stained with TRAP, and the cell number was determined.

## Slide 4
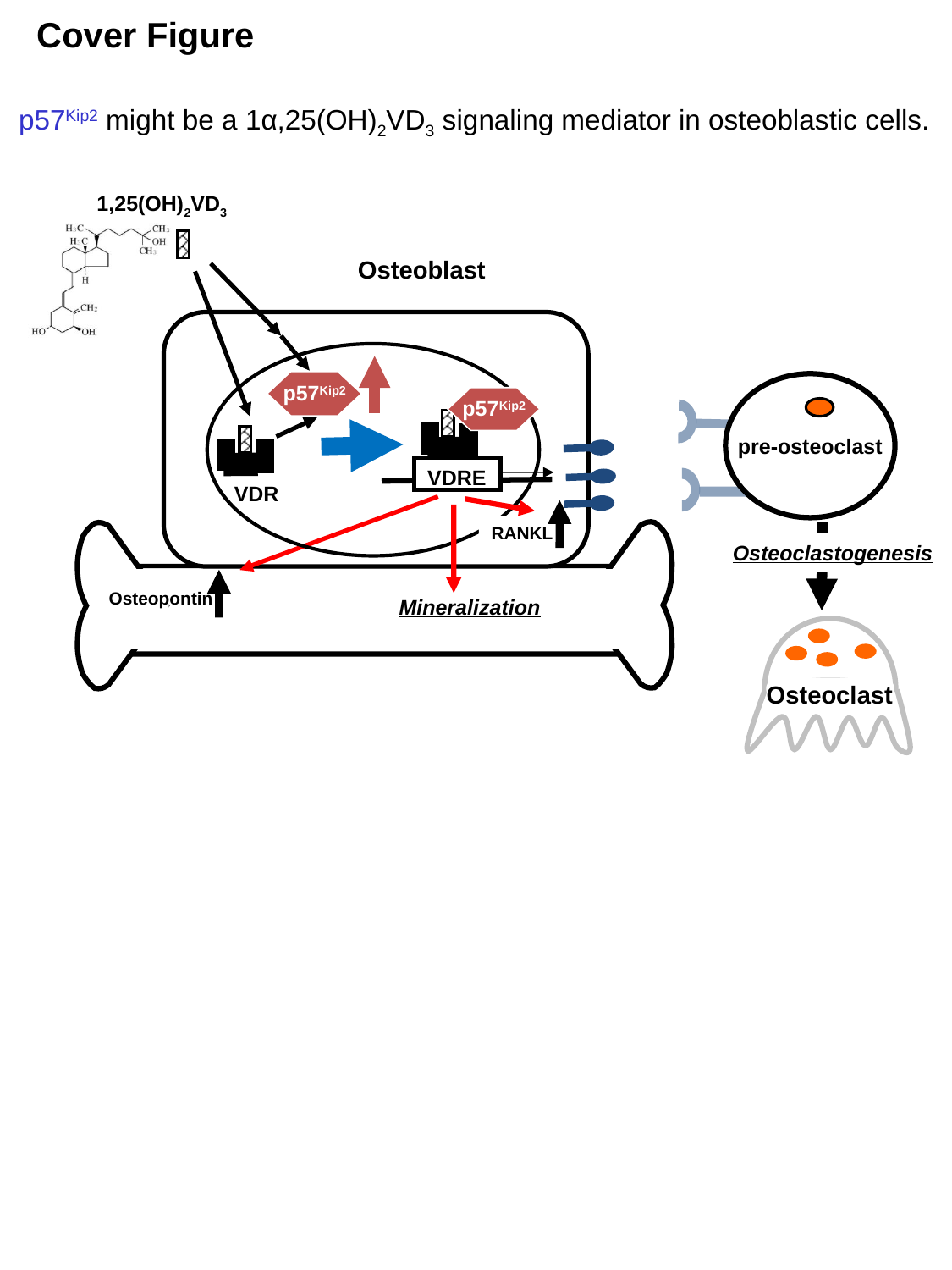

# Cover Figure
p57Kip2 might be a 1α,25(OH)2VD3 signaling mediator in osteoblastic cells.
1,25(OH)2VD3
Osteoblast
p57Kip2
pre-osteoclast
p57Kip2
 VDRE
VDR
RANKL
Osteoclastogenesis
Osteopontin
Mineralization
Osteoclast
