# Supplementary material for: p57Kip2 is an essential regulator of vitamin D receptor-dependent mechanisms
Source: PLoS One. 2023 Feb 15;18(2):e0276838. doi: 10.1371/journal.pone.0276838 (PMC9931147; doi:10.1371/journal.pone.0276838)
Supplement: Attachment — Submitted filename: Rebuttal_LETTER3_final.docx [file pone.0276838.s003.docx]

Review Comments to the Author

Reviewer #1: In the revised manuscript, the authors modified sentences and Figures, and responded to reviewer’s suggestions. However, there are still many mistakes and concerns in this manuscript.

1. Title still seems not to be suitable one, because this study seems to investigate role of p57 in 1, 25(OH)2D3 dependent transcription and functions in osteoblasts. Role of p57 in osteoclastogenesis is one of them.

Response

Thank you for your comment. We have never seen a reviewer who deeply understood our manuscript and figures in such detail, and this is a reasonable comment from the reviewers. For more than 30 years, we have only performed osteoclast research. As a result, our viewpoint became narrow, and the title was not appropriate. According to your indication, the title has been revised as "p57^Kip2^ is an essential regulator of vitamin D receptor-dependent mechanisms ".

2．Results; Page 16 line 304 to page 16 line 331: It is difficult to understand this paragraph. There are many mistakes in figure numbers. The authors should changes the order of Figures properly. Following figure numbers should be corrected (Fig 3A 3B to 3E (p16 line 312), Fig3D to 3C (page 17 line 320), Fig3E to 3D (page16 line 322) and

Response

We apologize for the many mistakes and concerns regarding this manuscript. We have corrected this issue accordingly.

3．Results; Page 19 line 365: D3 decreased RANKL ??? D3 stimulates RANKL in Fig 4A.

Response

We apologize for the many mistakes and concerns regarding this manuscript. We have corrected this issue accordingly. The correct term, upregulated, was added.

4．I could not find explanation of sentences as to Supplemental Figure 1, 3 and 4 in the manuscript. The authors should include explanation of these Figures in Results and Discussion.

Response

We apologize for the many mistakes and concerns regarding this manuscript. We have added explanations of Supplement Figure 1, 3, and 4 to the results section.

5. Discussion; Page 21 line 397, “In addition, p57kip2-deficient neonatal mice…” There are no data as to an osteomalacia-like phenotypes in this manuscript.

Response

We apologize for the many mistakes and concerns regarding this manuscript. We have deleted this paragraph.

Reviewer #2: Although this resubmitted manuscript almost is revised the pointed-out portions, there are some small problems yet. There are the concerns need to be addressed.

1) The re-submitted manuscript clearly clarified the regulation of osteoblast function, such as mineralization, proliferation, rankl(opn) expression. However, it is a major problem that the authors did not prove whether p57/kip2 promote osteoclastogenesis via increase rankl/opg ratio or not. Did the concerned paper publish? Did the authors examine the co-culture with p57/kip2 -/- primary osteoblast or p57 overexpression ST2 and bone marrow macrophages in the presence of vitamin D3? Otherwise, the authors should revise the phrase “regulator of osteoclastogenesis” in title of the manuscript.

Response

Thank you for your comment. We have never seen a reviewer who deeply understood our manuscript and figures in such detail, and this is a reasonable comment from the reviewers. According to your indication, the title has been revised as "p57^Kip2^ is an essential regulator of vitamin D receptor-dependent mechanisms ".

2) The authors indicated the promotion of VDR-p57 complex in rank expression. What do the authors speculate that the complex inhibited the expression of opg? Do the authors speculate the complex bind in the suppressor site of opg or not?

Response

Nakamichi Y et al reported that VDR in osteoblast-lineage cells primarily mediates the vitamin D treatment-induced increase in bone mass by suppressing bone resorption.

J Bone Miner Res 32:1297-1308, 2017. They suggested that VDR banded in the suppressor site of the opg promotor.

3) Figure 2: The labeled pluses (2A&2B) were stretched vertically and little bit strange. There is no Fig. 2D (page 15, lines 283&286: inserted in text).

Response

Thank you for your comment. We apologize for the many mistakes and concerns regarding this manuscript. Fig 2B included two graphs, which were correctly labeled as Fig 2 B and 2C. The old Fig 2C was the correct Fig 2D. The manuscript and legend were corrected.

4) Cover Figure: Did the authors check the effect of p57/kip2 on cell adhesion in osteoblasts?

Response

We speculated that VD_3_ might enhance the adhesion ability of osteoblasts through a p57-VDR interaction, but we had not studied this yet. The reviewer’s comments showed us the importance of performing an adhesion assay. When we attempted an adhesion assay using primary osteoblasts, we observed similar adhesion ability of WT and p57-null osteoblasts to the tissue culture plate. VD_3_ treatment did not significantly affect the cell adhesion of either WT or p57-null osteoblasts.

Katayama Y et al. also reported that recombinant rat osteopontin (OPN) enhances adhesion of osteoclasts but not osteoblasts and that specific phosphorylation of OPN is important for interaction with osteoclasts, compared with osteoblastic cells. Alternative integrin may be important in the interaction between osteoblastic cells. J Cell Physio 176(1):179-87. 1998. Our data supported Katayama’s report. Therefore, we deleted “cell adhesion” from our Cover Figure.

**Adhesion ratio**

**0**

**0.5**

**1**

**0.1% DMSO**

**10 nM VD3**

***p57+/+***

***p57-/-***

Editor’s comment

Figure 4

Please change the 3 in “VD3” to a subscript for consistency with the main text

Response

Thank you for your comment. We have corrected this issue.
